# Characterizing Neural Entrainment to Hierarchical Linguistic Units using Electroencephalography (EEG)

**DOI:** 10.3389/fnhum.2017.00481

**Published:** 2017-09-28

**Authors:** Nai Ding, Lucia Melloni, Aotian Yang, Yu Wang, Wen Zhang, David Poeppel

**Affiliations:** ^1^College of Biomedical Engineering and Instrument Sciences, Zhejiang University, Hangzhou, China; ^2^State Key Laboratory of Industrial Control Technology, Zhejiang University, Hangzhou, China; ^3^Key Laboratory for Biomedical Engineering of Ministry of Education, Zhejiang University, Hangzhou, China; ^4^Interdisciplinary Center for Social Sciences, Zhejiang University, Hangzhou, China; ^5^Neuro and Behavior EconLab, Zhejiang University of Finance and Economics, Hangzhou, China; ^6^Neuroscience Department, Max-Planck Institute for Empirical Aesthetics, Frankfurt, Germany; ^7^Department of Neurology, New York University Langone Medical Center, New York, NY, United States; ^8^Department of Neurophysiology, Max-Planck Institute for Brain Research, Frankfurt, Germany; ^9^Department of Psychology, New York University, New York, NY, United States; ^10^School of Computer and Information Engineering, Zhejiang Gongshang University, Hangzhou, China

**Keywords:** EEG, entrainment, speech, phrase, hierarchical structures

## Abstract

To understand speech, listeners have to combine the words they hear into phrases and sentences. Recent magnetoencephalography (MEG) and electrocorticography (ECoG) studies show that cortical activity is concurrently entrained/synchronized to the rhythms of multiple levels of linguistic units including words, phrases, and sentences. Here we investigate whether this phenomenon can be observed using electroencephalography (EEG), a technique that is more widely available than MEG and ECoG. We show that the EEG responses concurrently track the rhythms of hierarchical linguistic units such as syllables/words, phrases, and sentences. The strength of the sentential-rate response correlates with how well each subject can detect random words embedded in a sequence of sentences. In contrast, only a syllabic-rate response is observed for an unintelligible control stimulus. In sum, EEG provides a useful tool to characterize neural encoding of hierarchical linguistic units, potentially even in individual participants.

## Introduction

A critical feature of human language is that it can concatenate smaller units, e.g., words, into larger structures, e.g., phrases, and recursively bind such units into larger structures like sentences, governed by the constraints of a grammatical system (Chomsky, 1957; Fitch and Friederici, 2012; Berwick et al., 2013; Everaert et al., 2015). During speech comprehension, the acoustic speech signal is first mapped onto phonetic features, which are then deployed to retrieve lexical information (Poeppel et al., 2008). To understand sentences, words have to be further combined into phrases and sentences, based on tacit grammatical knowledge (Townsend and Bever, 2001; Phillips, 2003). A number of studies have investigated the cortical network involved in the process of combining words into phrases and sentences (Friederici et al., 2000; Lerner et al., 2011; Pallier et al., 2011; Nelson et al., 2017). Those studies have shown increased activation in a distributed network involving the inferior frontal gyrus and the superior and middle temporal gyri when words combine into phrases. In terms of the neurophysiological processes, studies have shown that when syllables combine into words, the first syllable in a word elicits larger electroencephalography (EEG) responses at latency of around 100 ms (Sanders et al., 2002) and that cortical activity tracks the rhythms of both syllables and words (Buiatti et al., 2009; Kabdebon et al., 2015; Farthouat et al., 2016; Batterink and Paller, 2017).

When investigating phrase-level neurophysiological processing, an EEG component, i.e., the closure positive shift (CPS), is observed at the boundary of prosodic phrases, which has been interpreted as a marker for phonological level processing of phrases (Steinhauer et al., 1999; Li and Yang, 2009). Recent magnetoencephalography (MEG) and electrocorticography (ECoG) experiments show that low-frequency cortical activity is concurrently entrained, i.e., synchronized, to the rhythms of multiple linguistic units, e.g., words, phrases, and sentences, even without any prosodic cues at the phrasal/sentential boundaries (Ding et al., 2016). Furthermore, within a linguistic structure, the power of electrophysiological activity shows a sustained increase or build up in the theta, beta (Bastiaansen et al., 2010; Bastiaansen and Hagoort, 2015; Ding et al., 2016), gamma (Peña and Melloni, 2012), and high-gamma bands (Ding et al., 2016; Nelson et al., 2017). Neural tracking in different frequency bands may reflect the neural coupling across frequencies (Lakatos et al., 2005; Canolty et al., 2006), but it has also been suggested that beta and gamma bands may preferentially process syntactic and semantic information (Bastiaansen and Hagoort, 2015; Ding et al., 2016). These results indicate that during listening to connected speech, the brain can construct phrasal/sentential structure purely based on grammatical cues and entrain cortical rhythms to track the rhythms of these internally constructed linguistic units.

**Figure 1:**
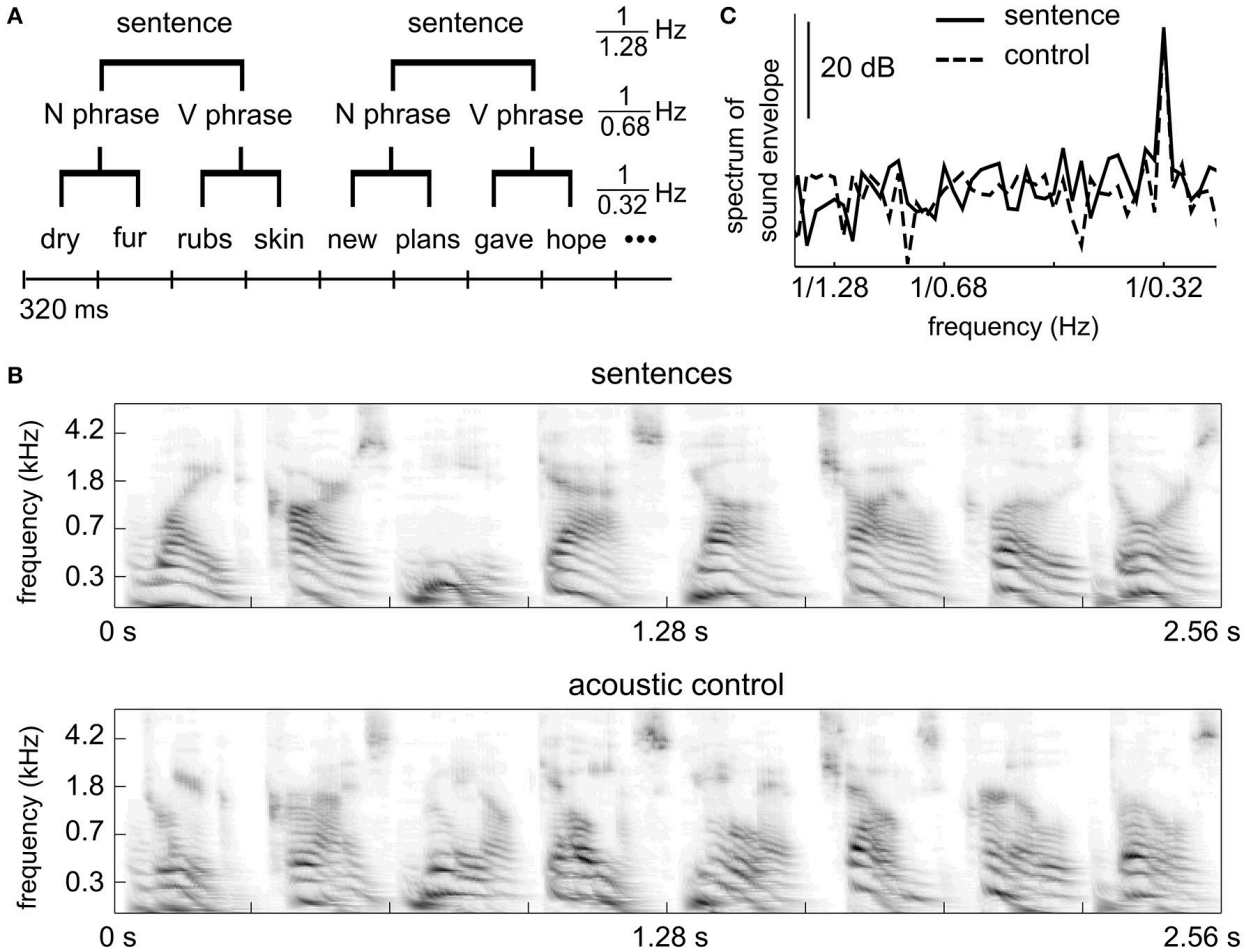
Stimulus. **(A)** Linguistic structure of the sentence stimuli. Each sentence contains a two-word noun phrase and a two-word verb phrase. All words are monosyllabic. **(B)** Auditory spectrogram of an example segment of the sentence stimulus (upper) and the control stimulus (lower). The acoustic control stimulus has spectro-temporal properties similar to the sentence materials. **(C)** Power spectrum of the stimulus envelope. Both the sentence stimulus and the control stimulus show temporal modulations at the syllabic rate, i.e., 1/0.32 Hz, but not at the phrasal or sentential rates.

Concurrent cortical entrainment to hierarchical linguistic units provides a plausible neural marker to characterize how linguistic structure building is affected by factors, such as attention and memory and also affords a measure to study developmental and aging effects on linguistic structure building. One limitation of this neural marker, however, is that it has only been validated using MEG and ECoG, which are not commonly available recording techniques. Here we test whether cortical tracking of hierarchical linguistic units (Figure 1A) can also be observed using EEG.

## Methods

### Subjects

Sixteen native American English speaking New York University undergraduate students (4 males; 2 left-handed; age range 18–22) participated in this study. They were given course credit for participation. The institutional review board of New York University approved the study protocol, and written informed consent was obtained from all participants before the study.

### Stimuli

The stimuli and procedures were identical to experiment 6 (sentence condition and shuffled sequence condition) of a recent MEG study (Ding et al., 2016). In the sentence condition, each English sentence contains 4 monosyllabic words (Figure 1A). The first two words form a noun phrase (adjective/pronoun + noun) and the last two words form a verb phrase (verb + noun). English words were synthesized independently using the MacinTalk Synthesizer (male voice Alex, in Mac OS X 10.7.5). Each monosyllabic word was adjusted to 320 ms in duration. In each trial, 12 sentences were presented without any acoustic gap between sentences (continuous, isochronous presentation). Therefore, each trial is 15.36 s in duration. Thirty trials were played, eight of which contained outliers. An outlier trial was the same as a normal trial except that 3 consecutive words from a roved position were replaced with 3 random words. The behavioral response was correct in 72% (*SD* = 9%) trial for the sentence condition.

In the control condition, each syllable in the sentence condition was cut into 5 slices (72-ms in duration with a 10-ms overlap between neighboring slices, which is smoothed by a 10-ms linear ramp) and slices at the same position within a sentence were shuffled across sentences. The control stimulus is not intelligible speech but has similar acoustic properties as the 4-syllable sentences (Figures 1B,C). Detailed procedures of how the control stimuli were made are described in Ding et al. (2016). The control stimulus has the same duration as the sentence stimulus and 30 trials were presented. Eight trials contained outliers consisting of 4 randomly chosen English words embedded in the stimulus. The behavioral response was correct in 81% (*SD* = 14%) of the trials for the control condition. The behavioral score of one subject (33%) clearly differed from other subjects. If this subject was excluded, the mean correct rate was 84% (*SD* = 6%).

The spectrogram of an example segment of a stimulus is shown Figure 1B for both conditions. The spectrogram is calculated using an auditory model (Yang et al., 1992). The power spectrum of the temporal envelope of the stimuli is shown in Figure 1C. The temporal envelope is the average of the spectrogram over frequencies, and the power spectrum is calculated using the Discrete Fourier Transform (DFT) without any smoothing window. The power spectrum in Figure 1C is averaged over all 15.36-s duration trials. The stimulus envelope shows a spectral peak only at the syllabic/word rate.

### Procedures

The experiment was conducted in a quiet room. The sentence condition and the control condition were presented in separate sessions and the order of these two sessions was counterbalanced over subjects. The participants were instructed to distinguish normal trials from outlier trials by pressing a response keys at the end of each trial.

### EEG recording

EEG was continuously recorded with a 128-channel EEG system (EGI, Inc., Eugene, OR), digitized at a sampling rate of 1,000 Hz (bandpass filter = 0.01–400 Hz) and referenced to the vertex (Cz). The impedance of electrodes was kept below 40 kO (Ferree et al., 2001). EOG artifacts were removed from the EEG recordings using ICA (Delorme and Makeig, 2004). Specifically, the 128-channel EEG signals were dimension reduced to 80 components using PCA and then the 80 principal components were further decomposed using ICA. An independent component was removed if in its topography the mean power over the most frontal 14 channels was more than 10 times stronger than the mean power over all other channels.

The EEG signal was lowpass filtered to 25 Hz, since the signals of interests are in the low-frequency region, at 1/1.28, 2/1.28, and 4/1.28 Hz. Data were re-referenced offline to a common average reference. The response to each trial was epoched. The recorded data from the first sentence of each trial was removed to avoid the transient EEG response to sound onset.

### Response power and inter-trial phase coherence

The EEG response in each trial was converted into the frequency domain using the DFT. After the first sentence was removed, each trial was 14.08 s in duration (9 sentences × 1.28 s/sentence) and therefore the frequency resolution of the DFT of the entire trial is 0.071 Hz, i.e., 1/14.08 Hz. If the DFT of the response in trial *k* is denoted as *X*_*k*_(*f*), the evoked power spectrum is shown in equation (1), where *K* is the total number of trials. *X*_*k*_(*f*) is complex-valued Fourier coefficient and is a function of frequency *f*. The evoked power reflects the power of EEG responses that are synchronized to the speech input. It is the same as the power spectrum of the EEG response waveform averaged over trials.

(1) $$E(f)=|\Sigma_{k}X_{k}(f){|}^{2}/K$$

The inter-trial phase coherence is defined in equation (2), where θ_*k*_ is the phase angle of each complex-valued Fourier coefficient, i.e., θ_*k*_ = <*X*_*k*_(*f*).

(2) $$R(f)={\left(\Sigma_{k}\text{cos}(\theta_{k})\right)}^{2}/K+{\left(\Sigma_{k}\text{sin}(\theta_{k})\right)}^{2}/K$$

The induced power, i.e., the power of EEG responses not synchronized to the speech input, is also calculated as the following formula, where <X(*f*)> denotes the mean over trials.

(3) $$I(f)=\Sigma_{k}|X_{k}(f)\;-<\text{​}X(f)\text{​}>\text{​}{|}^{2}/K$$

### Significance testing

The statistical significance of neural entrainment at a target frequency was tested for evoked power and inter-trial phase coherence, respectively. In the power test, to remove the 1/f trend of the response power spectrum, the response power at each frequency was normalized by the neighboring 14 frequency bins (7 bins on each side, which is equivalent to 0.5 Hz). The normalized power (equation 3), which can be viewed as a signal-to-noise measure, is:

(4) $$E_{n}(f)=E(f)/\Sigma_{\omega }E(\omega ),|\omega -f|<\text{​​}0.5\text{Hz},\omega \neq f$$

where ω denotes frequencies around the target frequency *f*.

In the phase coherence test, phase coherence values are not normalized by the neighboring frequency bins, since the inter-trial phase coherence spectrum has no 1/f fall-off.

For the power test, the null hypothesis is that the power at the target frequency is not significantly larger than the power in neighboring frequencies. Under the null hypothesis, the normalized power *E*_*n*_(*f*) is subject to an *F*_(32, 448)_ distribution for each channel. When the response power is averaged over channels, since the EEG response is correlated over channels, we conservatively assumes that the normalized power calculated based on the power averaged over channels is also subject to an *F*_(32, 448)_ distribution. For the phase coherence test, the null hypothesis is that the response phase is not synchronized to the stimulus and the null distribution of θ_*k*_ is a uniform distribution. Therefore, we employed the *F*-test and the Rayleigh test, respectively, to evaluate the statistical significance of the evoked power and phase coherence at each target frequency.

For the response averaged over channels, the null distribution of the evoked power or phase coherence cannot be easily described by a parametric distribution due to the correlation between channels. Therefore, the null distribution of normalized power (or inter-trial phase coherence) is estimated based on the response at non-target frequencies, i.e., the responses at frequencies that are not harmonically related to the sentential rate. The chance-level normalized power (or phase coherence) is pooled over frequencies. When the significance test is applied to individual subjects, the chance-level power (or phase coherence) is pooled over subjects. The statistical significance of the response at a target frequency is the probability that the target-frequency response differs from a chance-level response.

A linear classification analysis is employed to test if the topographic patterns at two frequencies or in two conditions are significantly different. In this analysis, the topographic plots averaged over half of the trials (e.g., the first or last 15 trials) are used to train a classifier. Each subject is viewed as a sample. The classifier's performance is evaluated based on the data averaged over the other half of the trials. A binomial test (*N* = 16, probability: 0.5) is used to test if the classifier can discriminate the two classes of topographic plots with higher than chance performance.

## Results

**Figure 2:**
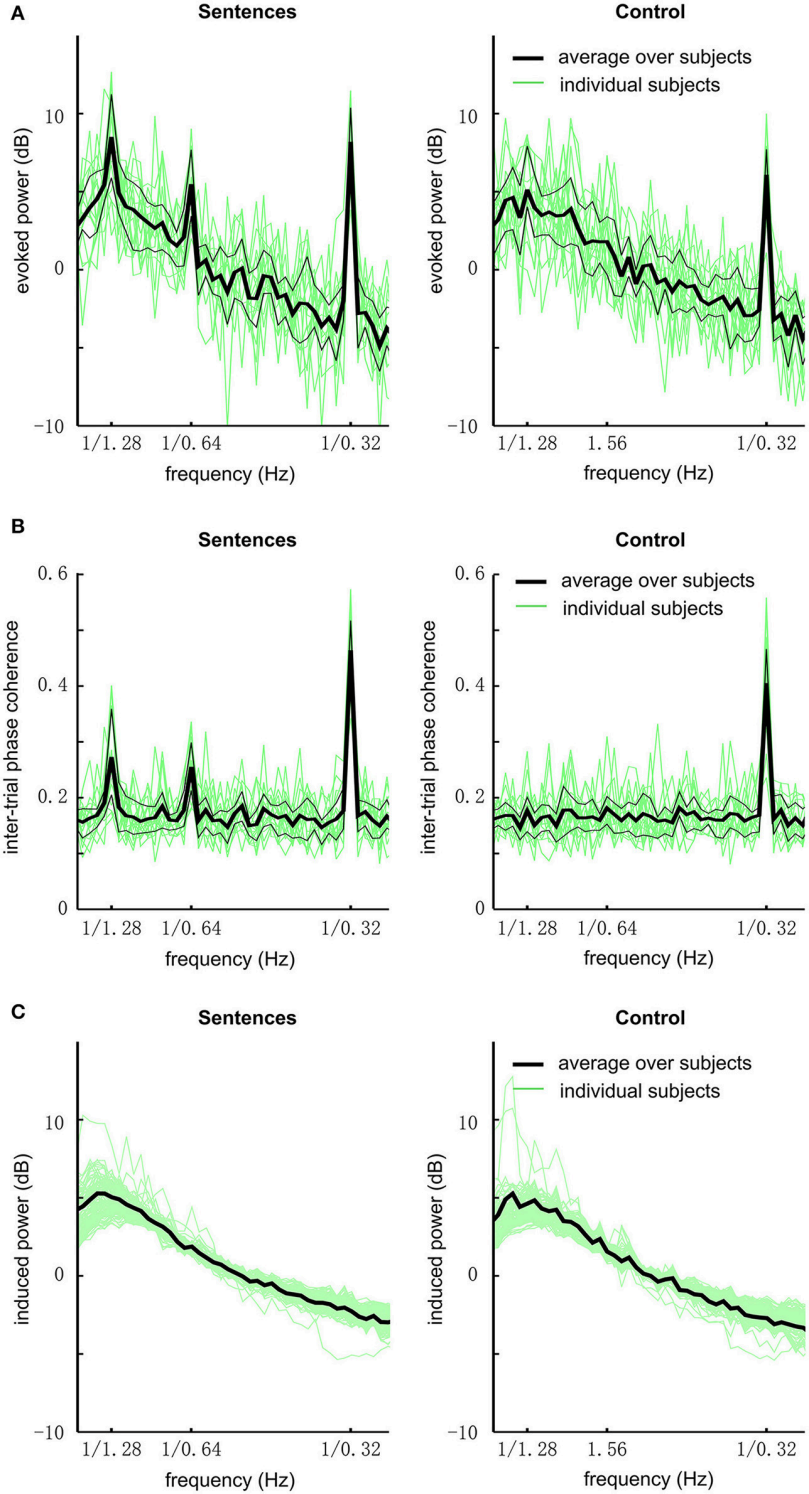
EEG responses to sentences and the acoustic control (grand average over all subjects and channels). **(A)** The evoked power spectrum of EEG responses. The bold black line shows the grand average over subjects, the two thin black lines delimit the 75th and 25th percentiles. Green lines show the data from individual subjects. The responses to sentences show 3 peaks at the sentential (1/1.28 Hz), phrasal (1/0.64 Hz), and syllabic rates (1/0.32 Hz), respectively. In contrast, the responses to the acoustic control only show one peak at the syllabic rate. **(B)** The spectrum of inter-trial phase coherence. The phase coherence spectrum is similar to the evoked power spectrum except that there is no 1/f power fall-off in the spectrum. Statistically significant inter-trial phase coherence means that the EEG responses are phase-locked to the stimulus. **(C)** Induced power.

We first analyzed the global field power of EEG responses (Figure 2A). In this analysis, the power spectrum is calculated for each electrode and then averaged over electrodes. In the grand average over subjects, the response to sentences shows three clear peaks at the sentential, phrasal, and syllabic rates, respectively [*P* < 0.001, *F*_(32, 448)_ = 8.9, 6.1, and 111.9, respectively]. The response to the acoustic control shows a single statistically significant peak at the syllabic rate [*P* < 0.001, *F*_(32, 448)_ = 47.6]. The response at the sentential and phrasal rates are not significantly stronger than the power in neighboring frequency bins [*P* > 0.3, *F*_(32, 448)_ = 1.1, and 1.1 respectively]. Comparing the sentence condition and the control condition, it is revealed that the response is stronger for the sentential condition at the sentential [*P* < 0.001, *F*_(32, 32)_ = 7.9], phrasal [*P* < 0.001, *F*_(32, 32)_ = 4.5], and syllabic rates [*P* = 0.03, *F*_(32, 32)_ = 2.0].

To quantify if the neural response is phase-locked to the stimulus, we calculated the inter-trial phase coherence. The inter-trial phase coherence averaged over channels is shown in Figure 2B. For the sentence condition, three peaks in the phase coherence spectrum are observed at the sentential, phrasal, and syllabic rates, respectively (*P* < 0.002, see Methods). For the control condition, only one peak at the syllabic rate is observed (*P* < 0.002).

The induced power, i.e., non-phase-locked power, of the EEG responses is shown in Figure 2C. No spectral peak is observed at the sentential, phrasal, or syllabic rate.

**Figure 3:**
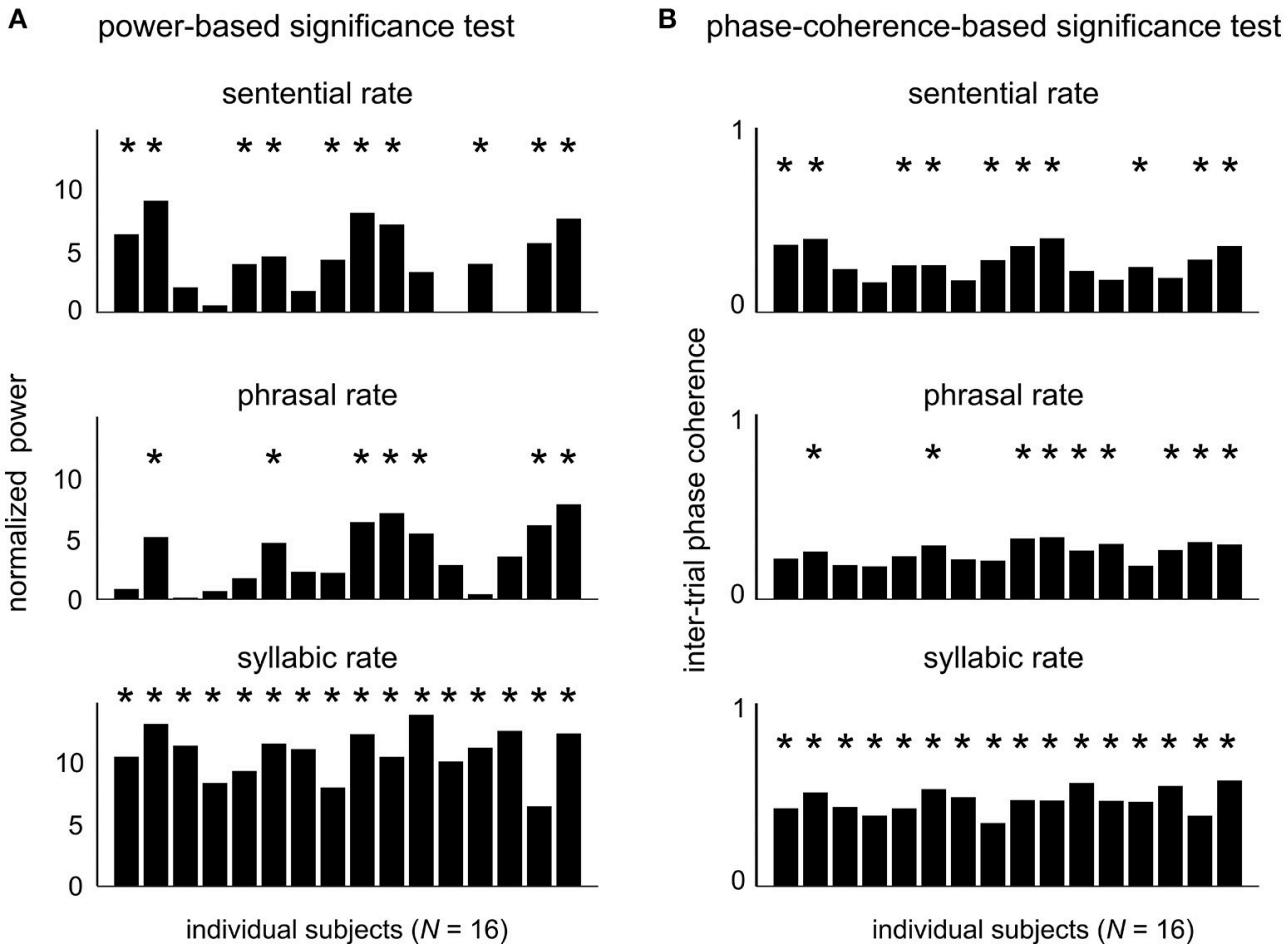
Neural responses to hierarchical linguistic structures in individual participants. The normalized evoked power **(A)** and inter-trial phase coherence **(B)** for individual subjects at each target frequency. Participants showing a statistically significant spectral peak at each target frequency are marked by a star (*P* < 0.05, FDR-corrected, see Methods).

The EEG responses in the sentence condition were further analyzed as follows. We first quantify whether the neural responses to hierarchical linguistic units can be reliably detected in single subjects. The response power from individual subjects is shown in Figure 3A for each target frequency. The power at each target frequency was normalized by the mean power in a 1-Hz neighboring frequency area (0.5 Hz on each side of the target frequency). At the sentential, phrasal, and syllabic rate responses reached significance level (*P* < 0.05, FDR-corrected, see Methods) in 62.5, 43.8, and 100% of the 16 participants, respectively. The inter-trial phase coherence values of individual subjects is shown in Figure 3B. Statistically significant phase coherence was observed at the sentential, phrasal, and syllabic rates in 62.5, 56.3, and 100%, respectively (*P* < 0.05, FDR-corrected, see method). Neural entrainment to at least one higher-level linguistic structure (i.e., phrase or sentence) is detected in 68.8% (*N* = 11) and 81.3% (*N* = 13) of the subjects for the power test and the phase test, respectively.

**Figure 4:**
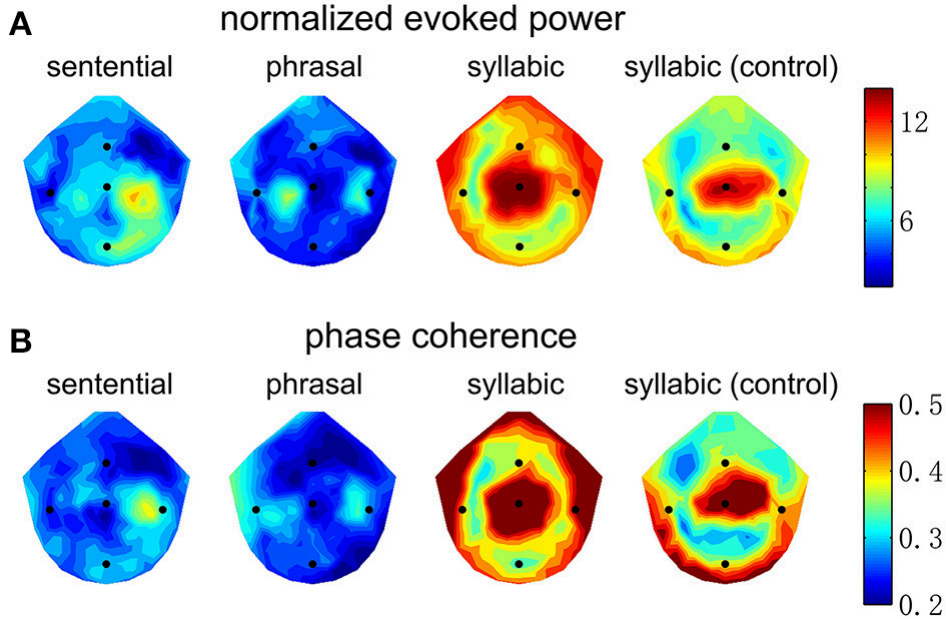
Topography of the neural responses at the sentential, phrasal, and syllabic rates. Channels Cz, T7, T8, Fz, and Pz are marked by blue dots. **(A)** The normalized evoked response averaged over subjects. The color bar shows the normalized power in dB. The syllabic rate response is strongest near channel Cz. In contrast, the sentential response is strongest between Cz and T8 and the phrasal response shows a bilateral pattern on both sides of Cz. **(B)** The inter-trial phase coherence averaged over subjects, shows a pattern similar to the that of normalized evoked power.

The spatial distribution of EEG power and phase coherence over electrodes is shown in Figure 4. The syllabic response is most salient around channel Cz. The sentential and phrasal rate responses, however, are more salient near channels on the right and left side of channel Cz. Such a distinction in spatial distribution, however, is not consistent across subjects, since a linear classifier fails to distinguish the topographic patterns between the conditions shown in Figure 4 above chance level, possibly due to the low-spatial resolution of EEG and large individual differences.

**Figure 5:**
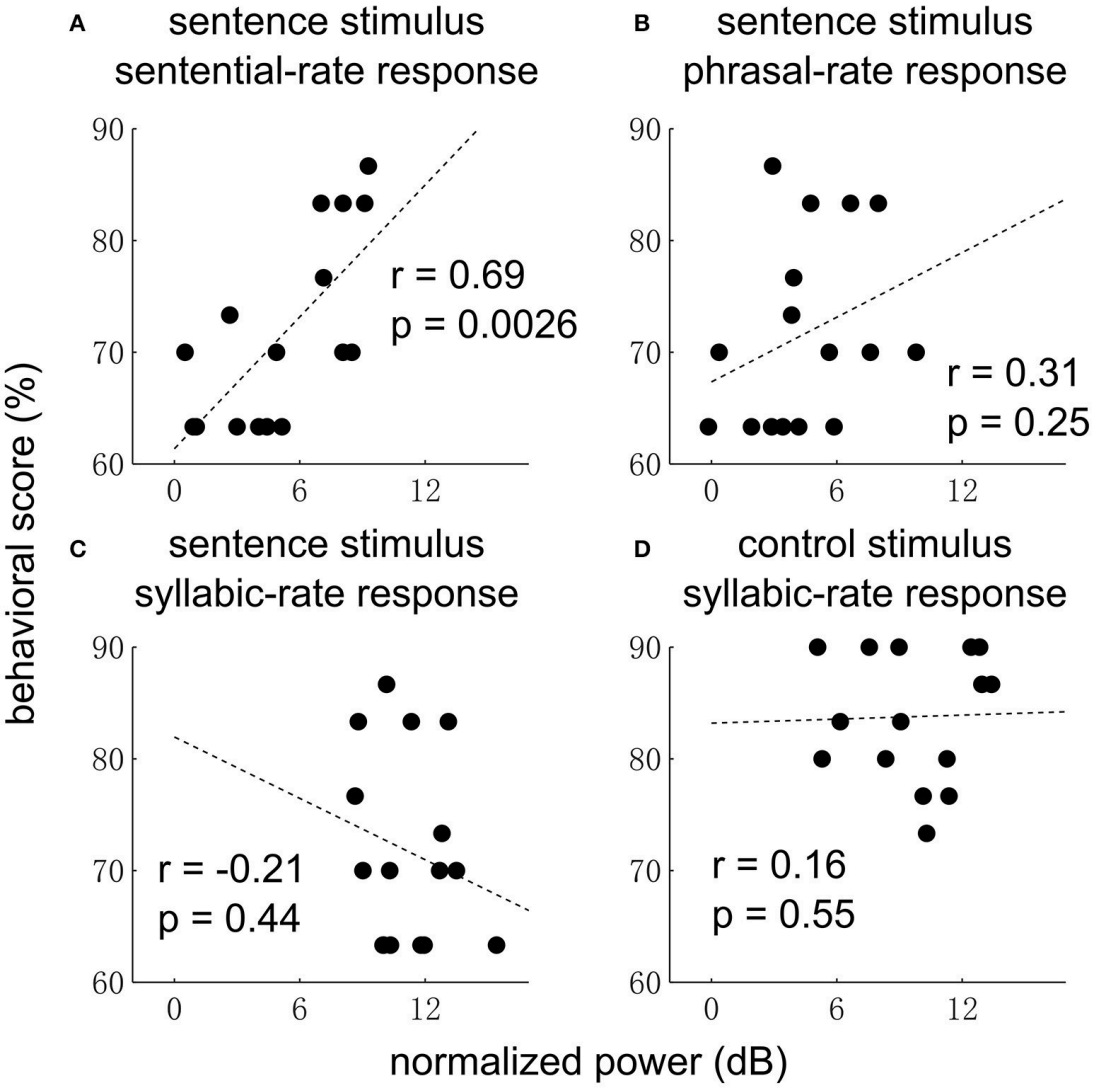
Correlation between neural response power and behavior for the sentence **(A–C)** and control condition **(D)**. In the sentence condition, the sentential-rate response is positively correlated with how well each subject can detect a sequence of random words embedded in a sequence of sentences. In **(D)**, data from one subject is not shown since the behavioral score (33%) is much lower than other subjects.

Finally, we examine whether the EEG responses are correlated with behavior (Figure 5). The sentential-rate response is found to be significantly correlated with the performance of detecting an outlier (i.e., 3 random words) embedded in a sequence of grammatical sentences. No correlation with behavior is observed at other frequencies.

## Discussion

This study shows that ongoing EEG responses can follow the rhythmic structure of multiple linguistic levels, concurrently, during listening to connected speech. These results demonstrate that scalp EEG provides an effective tool to investigate the neural tracking of hierarchical linguistic units in individual subjects.

### Neural representation of hierarchical linguistic units

Whether sentences are represented by hierarchically embedded syntactic structures or linear Markov models during language comprehension is a central question in cognitive science (Chomsky, 1957; Townsend and Bever, 2001; Jackendoff, 2002; Phillips, 2003; Frank et al., 2012; Everaert et al., 2015). Recently, a number of studies have suggested that neural processing of languages cannot be fully explained by a linear Markov model and is consistent with hierarchical syntactic models. For example, using fMRI, Brennan et al. tested how well BOLD signals in each cortical area can be explained by a hierarchical models or Markov models (Brennan et al., 2016). They found that while hierarchical models predicted BOLD signals in the anterior and posterior temporal lobes, a Markov model predicted activity in a broader cortical network including the frontal lobe. Neural entrainment to linguistic structures also provides a useful tool to address how syntactic structures are represented in the brain. For example, previous MEG data show that neural activity can entrain to linguistic structures even without any statistical cues for structural boundaries, demonstrating that statistical cues are not the only cues for syntactic analysis (Ding et al., 2016).

### Neural entrainment to speech

When listening to speech, cortical activity is entrained to the temporal envelope of speech (Luo and Poeppel, 2007; Kerlin et al., 2010; Lalor and Foxe, 2010), which carries the acoustic rhythm of speech. It has also been shown that cortical activity carries phonetic information (Di Liberto et al., 2015). Furthermore, as shown in this study using EEG and previous studies using MEG and ECoG (Ding et al., 2016), cortical activity is also entrained to the rhythms of higher level linguistic structures such as phrases and sentences, in the absence of acoustic cues. Therefore, during speech listening, cortical activity on different time scales is concurrently synchronized to linguistic structures of time scales. Therefore, a hierarchy of linguistic structures are converted into neural dynamics on different time scales, providing a plausible neural basis for the mental representation of hierarchical linguistic structures and the interactions between linguistic levels during speech processing (Townsend and Bever, 2001; Poeppel et al., 2008; Christiansen and Chater, 2016).

Although a syllabic-rate response is observed in both the sentence condition and the control condition, it is weaker in the control condition. This effect may reflect better neural tracking of intelligible monosyllabic words. Alternatively, it is possible that the subjects paid more attention in the sentence condition, which enhances the syllabic/word rate response. The sentence condition may require a higher level of attention, since the behavioral task is more challenging in the sentence condition (~70% correct rate in the sentence condition vs. ~80% correct rate in the control condition).

To dissociate syntactic from prosodic processing, the current study removes prosodic cues in speech. Natural speech, however, contains rich prosodic information which facilitates syntactic analysis. Therefore, neural tracking of phrasal and sentential structures is likely to be more prominent in natural speech. Furthermore, prosodic cues can also directly generate event-related response tracking the structural boundaries, such as the CPS (Steinhauer et al., 1999).

### Measuring neural tracking of phrases and sentences using EEG

In this study, 30 trials of sentence sequences (~15 s each) are presented and the neural tracking of higher-level linguistic structures, i.e., phrases or sentences, can be detected in more than half of the subjects using EEG. Therefore, the EEG-based paradigm shown here provides a plausible way to measure the neural encoding of higher-level linguistic structures. The sentential and phrasal responses do not reach significance in some subjects, possibly limited by the low SNR of EEG recordings. The subjects in the current study are all young adult native speakers without any language disorders, and therefore individual differences in language ability should be small, given such elementary processing demands. Nevertheless, the performance of detecting random words embedded in a sequence of sentences shows considerable individual differences, which is likely to be driven by cognitive factors, e.g., attention, rather than language ability. Indeed, a recent study shows that neural tracking of phrasal and sentential structure is diminished during sleep (Makov et al., 2017). Future experiments are needed to elucidate the influence of tasks and cognitive states on the neural tracking of phrasal and sentential structures.

It is challenging to detect of low frequency neural activity, since background neural activity generally has a 1/f spectrum. Compared with the 4-Hz syllabic-rate response and the 1-Hz sentential-rate response, however, the 2-Hz phrasal-rate response is especially difficult to detect in individual subjects. A possible reason is the following: The syllables have very clear acoustic boundaries and therefore can drive strong auditory responses. Each sentence is a syntactically and semantically coherent unit and, in this experiment, different sentences are syntactically and semantically disconnected. Therefore, the sentences also have relatively clear perceptual boundaries. The phrases within a sentence, however, are related both syntactically and semantically, which makes the boundaries between them less obvious than those between syllables and sentences.

Finally, since EEG is a commonly available non-invasive neural recording technique, the current paradigm has the potential of being developed into a tool to assess higher-level linguistic processing in populations less able to engage in typical laboratory research, including children and clinical patients. To apply the current paradigm to a special population, however, possibly requires adapting the sentence materials based on the vocabulary familiar to the target population and elucidating how attention and other cognitive factors may influence the neural tracking of higher-level linguistic structures.

## Author contributions

ND, LM, and DP conceived the experiment. AY performed the experiments. AY, YW, WZ, and ND analyzed the data. ND, LM, and DP wrote the paper. All authors edited the paper.

### Conflict of interest statement

The authors declare that the research was conducted in the absence of any commercial or financial relationships that could be construed as a potential conflict of interest.
